# Supplementary material for: Assessment of Complication Risk in the Treatment of Proximal Humerus Fractures: A Retrospective Analysis of 4019 Patients
Source: J Clin Med. 2023 Feb 25;12(5):1844. doi: 10.3390/jcm12051844 (PMC10003238; doi:10.3390/jcm12051844)
Supplement: Supplementary file 1 [file jcm-12-01844-s001.zip › jcm-2132447-supplementary.pdf]

Supplementary Materials:

Depiction of the number of patients per hospital, split into the proportion of patients with conservative or operative therapy, as well as the total number of patients.

Hospital	Operative treatment	Conservative treatment	Total number
1	158	105	263
2	360	172	532
3	497	58	555
4	282	172	454
5	314	87	401
6	496	261	757
7	313	101	414
8	156	96	252
9	289	102	391

Supplemental Table S1. Number of patients with conservative or operative treatment in each hospital.
